# Advances in nanotechnology-enabled adjuvants for peptide-based cancer vaccines

**DOI:** 10.26599/nr.2025.94907534

**Published:** 2025-05-22

**Authors:** Mengwen Li, Wenpan Li, Zhi Li, Jianqin Lu

**Affiliations:** 1 Skaggs Pharmaceutical Sciences Center, Department of Pharmacology & Toxicology, R. Ken Coit College of Pharmacy, The University of Arizona, Tucson, AZ 85721, USA; 2 Clinical and Translational Oncology Program, The University of Arizona Cancer Center, Tucson, AZ 85721, USA; 3 BIO5 Institute, The University of Arizona, Tucson, AZ 85721, USA; 4 Southwest Environmental Health Sciences Center, The University of Arizona, Tucson, AZ 85721, USA

**Keywords:** cancer vaccine, peptide-based vaccine, nanotechnology, adjuvants

## Abstract

Peptide-based vaccines only contain peptide epitopes and exclude unnecessary biological materials, which greatly reduces the risk of causing an undesired immune response and further improves the safety profile, garnering considerable interest in vaccine development. However, the immunogenicity induced by these peptides alone is not potent enough to elicit an effective immune response. Recently, combining the adjuvants with peptide antigens has shown promising effects to realize a satisfying immune response. In this review, we discuss the development of immunoadjuvants to enhance the safety and efficacy of peptide-based vaccines. The emphasis is placed on the application and clinical translation of nanotechnology-based adjuvants, highlighting the associated challenges and exploring future directions.

## Graphical Abstract

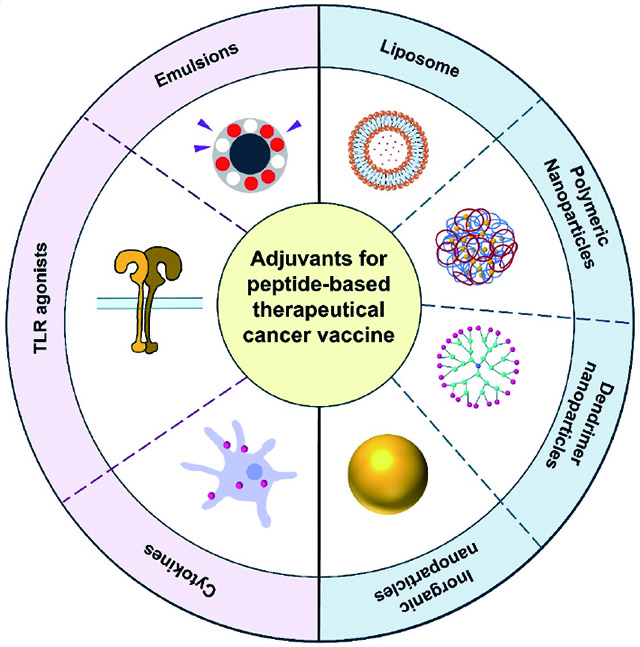

## 1 Introduction

Research on therapeutic vaccines for cancer has made tremendous progress over the past few decades and is now one of the most promising fields today in cancer treatment [1]. The history of cancer immunotherapy dates back to 1891 when Dr. William B. Coley injected inactivated strains of Streptococcus and Serratia (known as Coley’s Toxins) into patients with inoperable cancer. This pioneering approach aimed to improve patients’ conditions by stimulating their immune systems. The remarkable success in tumor regression following these injections laid a crucial foundation for the development of cancer immunotherapy [2, 3].

In recent years, various immunotherapy strategies, such as monoclonal antibodies, checkpoint inhibitors, and chimeric antigen receptor (CAR) T-cell therapy, have been approved for human use [4-6]. However, these approaches typically target a singular antigen and often fail to establish a long-lasting memory effect that could prevent tumor recurrence after treatment [7, 8]. Consequently, significant efforts have been dedicated to exploring novel immunotherapeutic modalities, particularly therapeutic cancer vaccines, which hold great potential to address the limitations associated with existing immunotherapeutic platforms.

Therapeutic cancer vaccines are designed to target tumors, eliminate residual cancer cells remaining after conventional treatments such as surgery, and generate a long-lasting anti-tumor memory to prevent recurrence by stimulating the patient’s adaptive immune system [9]. Peptide-based therapeutic cancer vaccines are among the simplest ways to elicit a highly targeted anti-tumor T-cell response [10]. These vaccines retain only the most essential parts of the antigen, minimizing unnecessary immune responses. This approach significantly reduces the potential risks of inducing autoimmune reactions or allergies, thereby greatly improving the vaccine's safety profile compared to other types of vaccines [11]. However, because of their simplicity, free antigenic peptides often exhibit poor stability and immunogenicity, which can hinder their ability to induce a sustained immune response. This limitation ultimately results in suboptimal vaccine efficacy [10]. As Scott et al. noted, even if scientists identify the most potent antigen, a critical issue remains: How to effectively present the antigen to the immune system [12]. Therefore, optimizing peptide antigens with adjuvants to boost immune responses is imperative. Based on their function, adjuvants can be classified into immunostimulants and antigen-delivery systems [13]. Because immunostimulants have been extensively summarized elsewhere [14], herein, we will briefly introduce classical immunostimulants typically used as adjuvants in peptide-based therapeutic cancer nanovaccines. We will mainly focus on deliberating the recent advances in nanotechnology-based adjuvants. Finally, we will discuss the clinical progress of cancer nanovaccines and identify the main roadblocks hindering their broader use, as well as future opportunities to overcome these challenges.

## 2 Immunopotentiators

As the name suggests, immunopotentiators are designed to enhance immune responses to an antigen [15]. By binding to pattern recognition receptors (PRRs), these agents can directly activate innate immunity, thereby amplifying the recognition and presentation of antigens [16]. When pathogen-associated molecular patterns (PAMPs) or damage-associated molecular patterns (DAMPs) bind to PRRs, they trigger the activation and maturation of antigen-presenting cells. This process leads to the secretion of pro-inflammatory cytokines and subsequently instructs adaptive immunity to present the antigen to T lymphocytes [17]. While the detailed mechanisms of immunopotentiators have been reviewed elsewhere [18], this section will primarily introduce typical immunopotentiators commonly used in the therapeutic peptide-based cancer vaccines.

### 2.1 Incomplete Freund's adjuvant (IFA)

IFA, administered as a water-in-oil emulsion, is commonly used in clinical trials for therapeutic cancer vaccines targeting breast cancer, cervical cancer, lung cancer, and melanoma [19-24]. Unlike complete Freund’s adjuvant (CFA), IFA omits unnecessary components such as heat-killed mycobacteria, which reduces the risk of acute granulomatous lesions at the injection spot. The mechanisms of IFA include: (1) the formation of a reservoir at the injection site that allows for the sustained release of the emulsified antigen over an extended period, and (2) the small size of the emulsion droplet promotes the uptake and presentation of antigens [25, 26]. As a result, IFA can induce robust immune responses in both humoral and cellular pathways [13].

Montanide incomplete Seppic adjuvant (ISA)-51 represents a modern generation of IFA that utilizes highly purified mineral oils, further reducing injection site toxicity [27]. Montanide ISA-51 is licensed for human use in a therapeutical cancer vaccine CimaVax-EGF, which targets stage IIIB/IV non-small-cell lung carcinoma (NSCLC) in Cuba [28]. In addition, SurVaxM is also formulated with Montanide ISA-51. This vaccine is undergoing Phase 2 clinical trial and is expected to treat glioblastoma [29].

### 2.2 Toll-like receptors (TLRs) agonists

In the past few years, there has been growing interest in exploring the potential of TLRs to enhance immune responses. TLRs represent the most prominent family of PRRs, with ten identified in humans (TLR1-TLR10) [30]. Among them, TLR1, TLR2, TLR4, TLR5, and TLR6 recognize microbial membrane components on the cell surface. In contrast, TLR3, TLR7, TLR8, and TLR9 are located intracellularly and mainly recognize nucleic acids [18, 31]. Notably, the agonists for TLR3, TLR7, TLR8, and TLR9 — such as polyinosinic: polycytidylic acid (poly I:C), resiquimod, imiquimod, and CpG — have garnered significant attention in preclinical and clinical studies [32]. For instance, a 5% imiquimod cream has been approved for the treatment of basal cell carcinoma [33]. Interestingly, Seung et al. recently developed a nanoliposome-based kinetically activating nanoadjuvant (K-nanoadjuvant). K-neoadjuvant dynamically combines two innate immune stimuli (TLR3a and timely activation of TLR7/8a), demonstrating exceptional anticancer efficacy while preventing immune cell exhaustion [34]. The detailed toll-like receptor-related clinical trials are summarized in Table 1.

### 2.3 NOD-like receptors (NLRs) agonists

NLRs are a family of pattern recognition receptors that play a key role in the innate immune system by detecting PAMPs and danger signals. Agonists of NLRs can help boost vaccine efficiency by improving antigen presentation and stimulating a more robust and effective immune response against tumor antigens [45]. Furthermore, it has been shown that NLRs-mediated peptidoglycan recognition exhibits a synergistic effect on TLRs. As a result, NLRs can cooperate with complementary TLRs agonists to achieve better adaptive immune response [46, 47]. A prime example of an NODlike receptor agonist is muramyl dipeptide (MDP) [47].

### 2.4 Stimulator of interferon genes (STING) agonists

STING is an intracellular receptor located in the endoplasmic reticulum that can produce cytokines, like type I interferon (IFN), by activating the cGAS-STING signal pathway [48]. The STING pathway is of significant interest in the field of immunotherapy, particularly in cancer treatment, as it can be harnessed to enhance the immune system's ability to recognize and attack tumors [49]. STING agonists are capable of promoting the maturation of dendritic cells, T cell responses, and humoral immune responses [50]. Consequently, they are being developed as adjuvants in cancer vaccines to stimulate a more potent immune response against cancer cells. Currently, STING agonists such as ASA404, ADU-S100, MK-1454, IMSA101, SB 11285, MK-2118 and E7766 are under clinical investigations [51]. However, poor pharmacokinetics and off-target toxicity limited their applications, and none have been licensed for cancer vaccine studies [50]. Therefore, numerous delivery systems have been developed for STING agonists to enhance the stability and prolong the retention time and show promise in preclinical studies [48, 52].

### 2.5 Interleukin-2 (IL-2) and granulocyte-macrophage colony-stimulating factor (GM-CSF)

Both IL-2 and GM-CSF belong to cytokines. IL-2 is a cytokine that can be combined with cancer vaccines to improve the treatment of human malignancies [53]. One of the mechanisms of IL-2 being adjuvant is to enhance the expansion and/or activation of T cells primed by vaccination [54]. It also plays an important role in regulating tolerance through regulatory T cells (T reg cells) [55]. IL-2 marked a milestone as the initial immunotherapy to gain approval for cancer treatment. This therapy has been sanctioned for combating metastatic renal cell carcinoma and metastatic melanoma [56].

GM-CSF, a potent cytokine activator, belongs to the family of hematopoietic cytokines and is generated by a range of cell types, such as T cells, B cells, macrophages, mast cells, endothelial cells, fibroblasts, and varieties of cancer cells [57]. Its primary role is to bolster the functions of antigen-presenting cells (APCs) by promoting the maturation, activation, and recruitment of dendritic cells (DCs), macrophages, monocytes, and eosinophils [58]. GM-CSF has been investigated as an adjuvant in multiple cancer vaccination trials, encompassing breast cancer, pancreatic cancer, colon cancer, melanoma, and prostate cancer [59].

Overall, the mechanism and clinical status of immunopotentiators are summarized in Table 2. Some immunopotentiators, like IL-2 and TLR agonists, are already licensed for human use or in clinical trials for cancer, others, like STING agonists and NLR agonists, are still under investigation, with ongoing research exploring their potential in combination therapies.

## 3 Nanotechnology-based adjuvants

Although there have been improvements in the vaccine when co-administering peptide antigens with immunopotentiators, conventional adjuvants do not satisfy the criteria of clinical trials for therapeutical cancer vaccines. In particular, conventional immunostimulants, such as IFA, cause localized reactogenicity at the injection site, including pain, inflammation, and swelling to more severe reactions, the formation of granulomas, abscesses, and skin ulceration. Adjuvants based on PAMP, like small-molecule TLR7/8 agonists (imiquimod and resiquimod), may induce pronounced adjuvant-associated systemic toxicity. In addition to mild symptoms such as fever and diarrhea, they may increase the risk of autoimmune diseases, cause chronic organ toxicity and immune bias, etc., due to aberrant immune activation [64-66]. Furthermore, traditional adjuvants may be easily degraded, leading to dissatisfaction in achieving specificity and strong potency [67, 68]. Consequently, despite the success of some immunopotentiators in early clinical trials, developing safe and potent adjuvants remains a significant challenge that warrants further exploration [69]. Recent advancements in nanotechnology have enabled researchers to extend endeavors in the rational design of adjuvant systems while maximizing the efficacy of immunostimulants [70].

Unlike conventional immunostimulants that only enhance antigen immunogenicity, nanoadjuvants offer improved outcomes in safety, efficacy, and patient compliance [5, 13, 71]. In this section, we will first explore the general advantages of nanotechnology-based adjuvants, followed by examples of applications to illustrate these points. At the end of this section, a summary table will be provided to clarify the highlights.

### 3.1 Advantages of nanotechnology-based adjuvants

Nanotechnology-based adjuvants offer several advantages over conventional adjuvants in cancer vaccines. These benefits improve efficacy, safety, and patient compliance, making them a promising option for cancer immunotherapy.

Nanotechnology-based adjuvants enable the precise delivery of the vaccine components, such as antigens and adjuvants, to targeted cells or tissues, including tumor sites or lymph nodes. This targeted delivery reduces the exposure of off-target organs to these substances, thereby minimizing unwanted side effects and improving the overall safety profile of the vaccine [72-74]. Additionally, nanocarriers enable the controlled release of antigens and adjuvants over time, leading to a more sustained and robust immune response compared to conventional adjuvants. This approach can result in stronger long-term immunity and the potential for using lower vaccine doses [75-77]. Nanotechnology-based adjuvants also protect peptide antigens and conventional adjuvant. Encapsulation within nanocarriers ensures the stability of these substances, enhancing their effectiveness by preventing degradation before stimulating the immune system [78].

Many cancers exhibit a "cold" tumor microenvironment (TME), which is immunosuppressive and hinders effective immune responses. Nanotechnology-based adjuvants can address this challenge by modifying the TME by enhancing antigen presentation, promoting dendritic cell activation, and stimulating effector T cell infiltration. Nanovaccine-induced immunogenic cell death within the TME leads to the release of tumor-associated antigens and danger signals. This process stimulates antitumor immune responses, enhances the recognition and elimination of tumor cells by the immune system, and contributes to tumor regression and the establishment of long-term immune memory [79, 80]. Furthermore, nanotechnology-based adjuvants enabled the combination with other therapeutics, also facilitating the co-delivery of multiple agents (e.g., antigens and adjuvants). This approach provides a more comprehensive and synergistic therapeutic strategy [81-83]. It is worth noting that some nanomaterials can act as selfa-djuvants, enhancing the immune response without the need for additional adjuvants. This not only simplifies vaccine formulation but also makes the immune response more efficient [84].

From the perspective of patient compliance, nanoadjuvants offer greater flexibility in vaccine administration. For example, non-invasive routes such as inhaled or topical formulations are designed for patients who may be uncomfortable with traditional injection methods. These personalized approaches extensively enhance patient adherence and comfort [85-87]. Additionally, due to controlled release and targeted delivery, a lower dose and fewer administrations are required to achieve an equivalent immune response. The reduced need for frequent dosing significantly improves patient compliance, leading to better therapeutic outcomes [88].

In summary, nanotechnology-based adjuvants offer distinct advantages over conventional adjuvants in terms of targeting delivery, controlled release, enhanced stability, and reduced side effects. Additionally, some nanomaterials can function as self-adjuvants, enhancing the immune response. The benefits of nanotechnology-based adjuvants are summarized in Table 3. In the next section, we will focus on the applications to provide examples illustrating these advantages.

### 3.2 Applications of nanoadjuvants in tumor therapy

#### 3.2.1 Liposome

In 1974, Allison and Gregorjiadis first reported using liposomes as immunological adjuvants [89]. Since then, interest in liposome-based peptide vaccines has surged [90]. Liposomes are concentric sphere vehicles composed of phospholipid bilayers and an inner hydrophilic core, allowing for the encapsulation of hydrophobic and hydrophilic cargo. With the shield of phospholipid bilayers, encapsulated cargo is prevented from degradation. Moreover, the fundamental properties, such as composition, size, and zeta potential, can be easily modulated to enhance antigen uptake by dendritic cells. Combined with biocompatibility and low toxicity, liposomes are considered ideal and versatile carriers for personalized vaccines [91].

The primary limitation of current peptide-based vaccines is the adverse events associated with IFA and Montanide ISA-51 in clinical trials, as well as their limited ability to effectively generate CD8 T cells *in vivo* [92, 93]. In recent decades, numerous liposome-based cancer vaccines have been developed to address this challenge and demonstrate the clinical translation potential of liposome applications in cancer treatment. For instance, Varypataki et al. demonstrated the efficacy of cationic liposome-based synthetic long-peptide (SLP) vaccines in eliminating established melanoma and human papillomavirus (HPV)-induced tumors. To detail, SLP liposomes were simply prepared by thin-film dehydration-rehydration method with 1,2-dioleoyl-3-trimethylammonium propane (DOTAP) and 1,2-dioleoyl-sn-glycero-3-phosphocholine (DOPC). The complete protection provided to mice against subsequent tumor attacks highlighted the promising potential of liposomal-SLP vaccines as therapeutic agents in the subcutaneous melanoma model [94]. Additionally, Shariat et al. reported on a liposomal peptide vaccine for breast cancer. In their study, the P5 peptide, which contained a major histocompatibility complex (MHC) class I restricted multi-epitope derived from the rat human epidermal growth factor receptor 2 (HER2)/neu protein, was conjugated with Maleimide-PEG2000-DSPE and incorporated into liposomes along with the adjuvant monophosphoryl lipid A (Lip-DOPE-P5-MPL). Compared to other formulations, Lip-DOPE-P5-MPL induced a higher level of IFN-*γ* production in CD8^+^ lymphocytes, more effectively inhibited tumor growth in the TUBO tumor mouse model, and significantly prolonged survival time [95].

**Figure 1:**
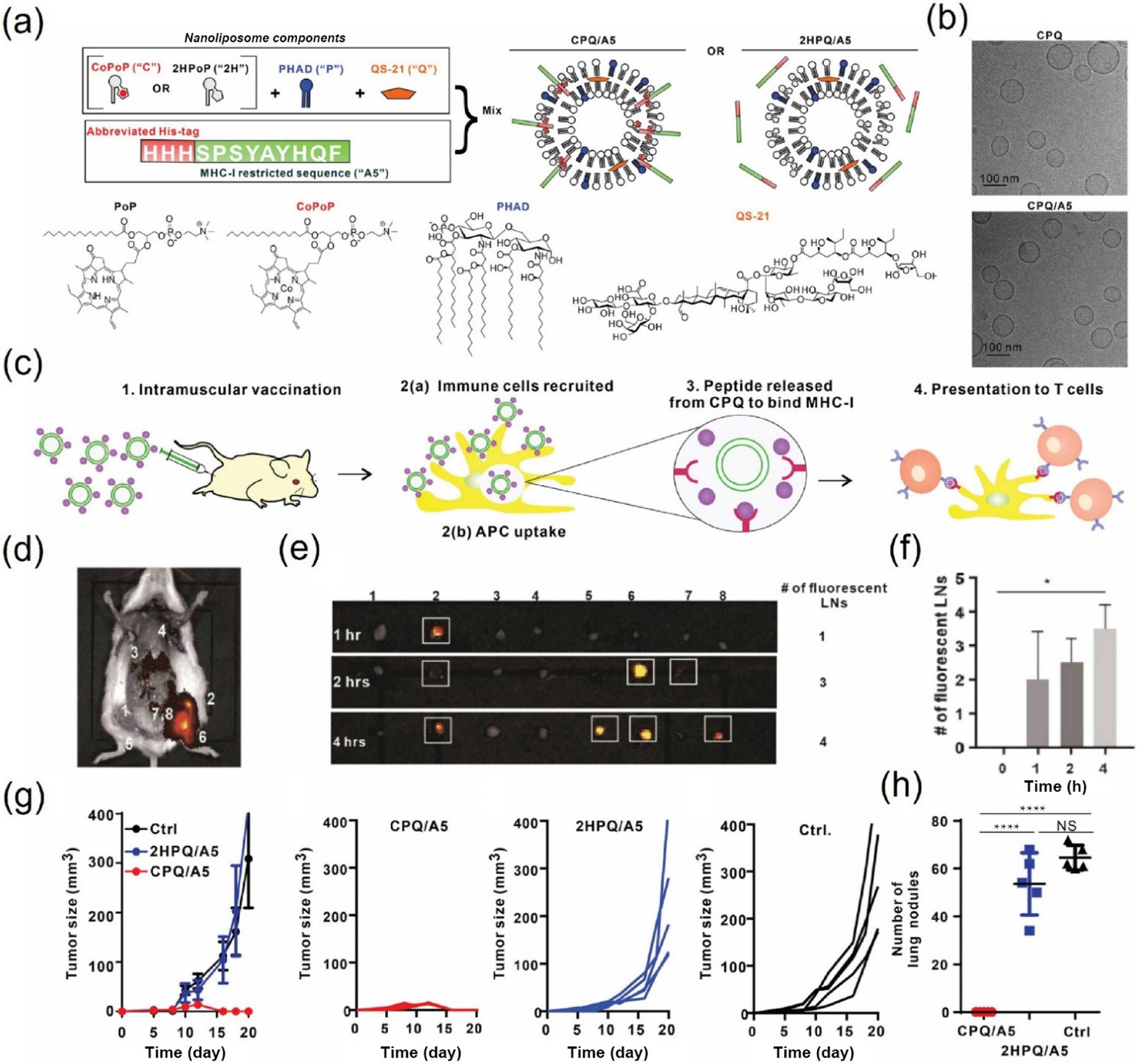
Characterization of CPQ/A5 and 2HPQ/A5. (a) Compositions of nanoliposome. (b) Cryo-electron micrographs of CPQ and CPQ/A5. (c) Schematic illustration of CPQ/A5 immunization. (d, e) Fluorescent imaging of liposome-drained lymph nodes. (f) Numbers of liposome-drained lymph nodes. (g) Therapeutic efficacy of 2HPQ/A5 and CPQ/A5 in the subcutaneous CT26 mouse model. (h) Numbers of lung metastasis nodules in the CT26 metastasis model after immunization [96]. Reproduced with permission from Ref. [96], © American Chemical Society 2021.

To address the incapability of short MHC-I-restricted peptides generating sufficient and continuous potent antigen-specific T cell response, liposomes containing cobalt porphyrin–phospholipid (CoPoP) binding with short MHC-I-restricted peptides, along with PHAD (synthetic monophosphoryl lipid A), and QS-21 was designed, in term CPQ/A5. Protected by the liposome, the short peptide antigen remained stable in serum and was efficiently transported to the draining lymph nodes, significantly enhancing uptake by antigen-presenting cells (APCs) and resulting in improved T cell activation. After the second CPQ/A5 liposome immunization, all tumors became smaller or disappeared in the CT26 tumor implantation model. Similarly, in the experimental lung metastasis model, no lung nodules could be observed in CPQ/A5 liposome-immunized mice, in stark contrast to more than 50 lung nodules in the control group, demonstrating the key role of liposomes in the therapeutic cancer vaccine (Fig. 1) [96].

Beyond the preclinical success, promising results of liposomal cancer vaccine have been seen in clinical trials [39, 97-101]. Notably, PDS0101 (Versamune^®^ HPV) is an HPV-16 E6/E7 multipeptide vaccine. It comprises the cationic lipid R-enantiomer of 1,2-dioleoyl-3-trimethylammonium-propane chloride (R-DOTAP) and HPV-16 E6/E7 peptides. Designed with a size comparable to viruses, it facilitates dendritic cell uptake. According to the company's white book on Versamune^®^, approximately 80% of dendritic cells can be detected with Versamune^®^ within 4 h following a subcutaneous injection.

In a completed Phase 1 study of PDS0101 monotherapy, the treatment demonstrated outstanding safety outcomes while inducing a high level of active HPV-specific CD8^+^ T cells [22, 102, 103]. To date, multiple Phase 2 studies combining PDS0101 with other treatments are on track, expecting to demonstrate significant disease control [104, 105]. For instance, the combination of PDS0101 and Pembrolizumab has proven safe and well-tolerated [104]. In the Phase 2 study, this combination significantly extended the median progression-free survival to 10.4 months, compared to the 2–3 months typically observed with approved immune checkpoint inhibitor monotherapies [106].

In the clinical study evaluating PDS0101 in combination with cisplatin and radiation therapy (NCT04580771), 87.5% of patients in the IMMUNOCERV group achieved a complete response at 3 months, with a 100% overall survival rate at one year. This is in contrast to the standard chemoradiation group, which showed no significant change [107]. Furthermore, the investigational combination altered the TH1-predominant cytokine profile, enhancing killer T cell activity and improving the polyfunctionality of T cells with enhanced killing function traffic to tumors [107]. Recently, the PDS Biotechnology company initiated a Phase 3 study of Versamune^®^ HPV for HPV16^+^ recurrent/metastatic head and neck squamous cell carcinoma. This marks the first-ever Phase 3 trial for HPV16^+^ head and neck cancer [108]. The details of the clinical trial of PDS0101 are summarized in Table 5.

Overall, the biocompatibility, biodegradability, and versatile encapsulation properties of liposome-based cancer vaccines contribute to their success in addressing safety concerns and generating effective CD8 T cell responses in preclinical studies (Table 4). In clinical trials, PDS0101 (Versamune^®^ HPV) has demonstrated promising safety and efficacy outcomes, with ongoing Phase 2 and 3 studies exploring its potential in combination with other treatments (Table 5). These advancements underscore the strong potential of liposomal cancer nanovaccines for clinical translation in cancer therapy.

#### 3.2.2 Polymeric nanoparticles

As mentioned before, rapid enzymatic degradation and low immunogenicity are two major drawbacks of antigenic peptides. To address these issues, polymeric nanoparticles have emerged as promising strategies. On the one hand, polymeric nanoparticles can be used as carrier systems to enhance the delivery efficiency of antigens and immunostimulatory molecules; on the other hand, some polymers are specifically designed to possess adjuvant functions that complement antigenic peptides [113]. So far, varieties of polymers have been explored for novel vaccine design. Among these, chitosan, poly(*ε*-caprolactone) (PCL), poly(lactide acid) (PLA), polystyrene, and poly(lactic-co-glycolic acid) (PLGA) have been approved by the U.S. Food and Drug Administration (FDA) due to their excellent safety profile, biodegradability, and biostability [114]. To address the limitations and adverse effects associated with IFA or Montanide ISA-51—commonly used in clinical trials involving HPV E6 and E7 peptides—which can cause severe local swelling, itching, redness, and pain [93, 115], Ma's group developed an innovative biodegradable PLGA nanoparticle system encapsulating the antigenic peptide from HPV16 E7 peptide (E744–62) and a novel adjuvant component, adenosine triphosphate (ATP). The ATP + E7-NPs significantly enhanced the *in vivo* stability of the E7 peptide while promoting dendritic cell uptake and lymph node accumulation of the antigen. Furthermore, potent antitumor effects were demonstrated in an HPV-associated mouse grafted tumor model. Following immunization, the growth of established tumors was significantly inhibited, which was mechanistically supported by elevated frequencies of CD8^+^ T cells and a decrease in the generation and tumor infiltration of immunosuppressive cells, including myeloid-derived suppressor cells and regulatory T cells. This indicated that ATP+E7-NPs may represent a promising therapeutical cancer vaccine candidate for clinical malignancy treatment [116].

**Figure 2:**
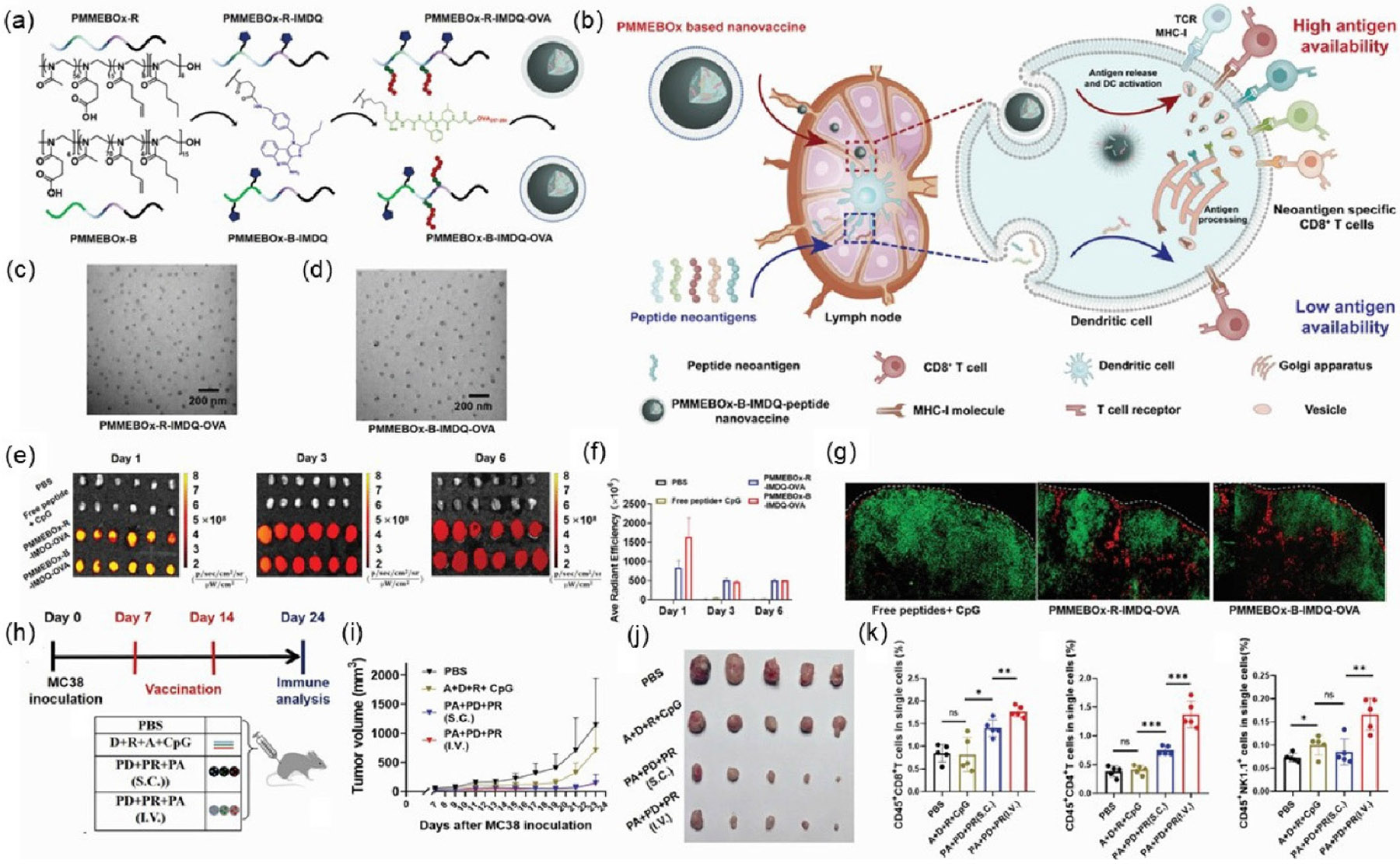
PMMEBOx-based nanovaccine improved the accumulation and infiltration of peptide antigens at lymph nodes, resulting in higher antigen availability. (a) Structures of PMMEBOx-R-IMDQ-OVA and PMMEBOx-B-IMDQ-OVA. (b) Schematic illustration of POx-based delivery system. (c) and (d) Transmission electron microscopy (TEM) image of PMMEBOx-R-IMDQ-OVA and PMMEBOx-B-IMDQ-OVA. (e) and (f) *Ex vivo* fluorescence and statistical analysis of vaccine-drained lymph nodes. (g) *Ex vivo* fluorescence of draining inguinal LN section after 24 h immunization. (h) Therapeutic scheme of PMMEBOx-B-IMDQ-neopeptide nanovaccine. (i) and (j) Tumor growth and images on MC38 trmor-bearing mice model. (k) Quantification analysis of CD8^+^, CD4^+^ T cell and natural killer (NK) cell after three doses of vaccination [113]. Reproduced with permission from Ref. [113], © American Chemical Society 2024.

**Figure 3:**
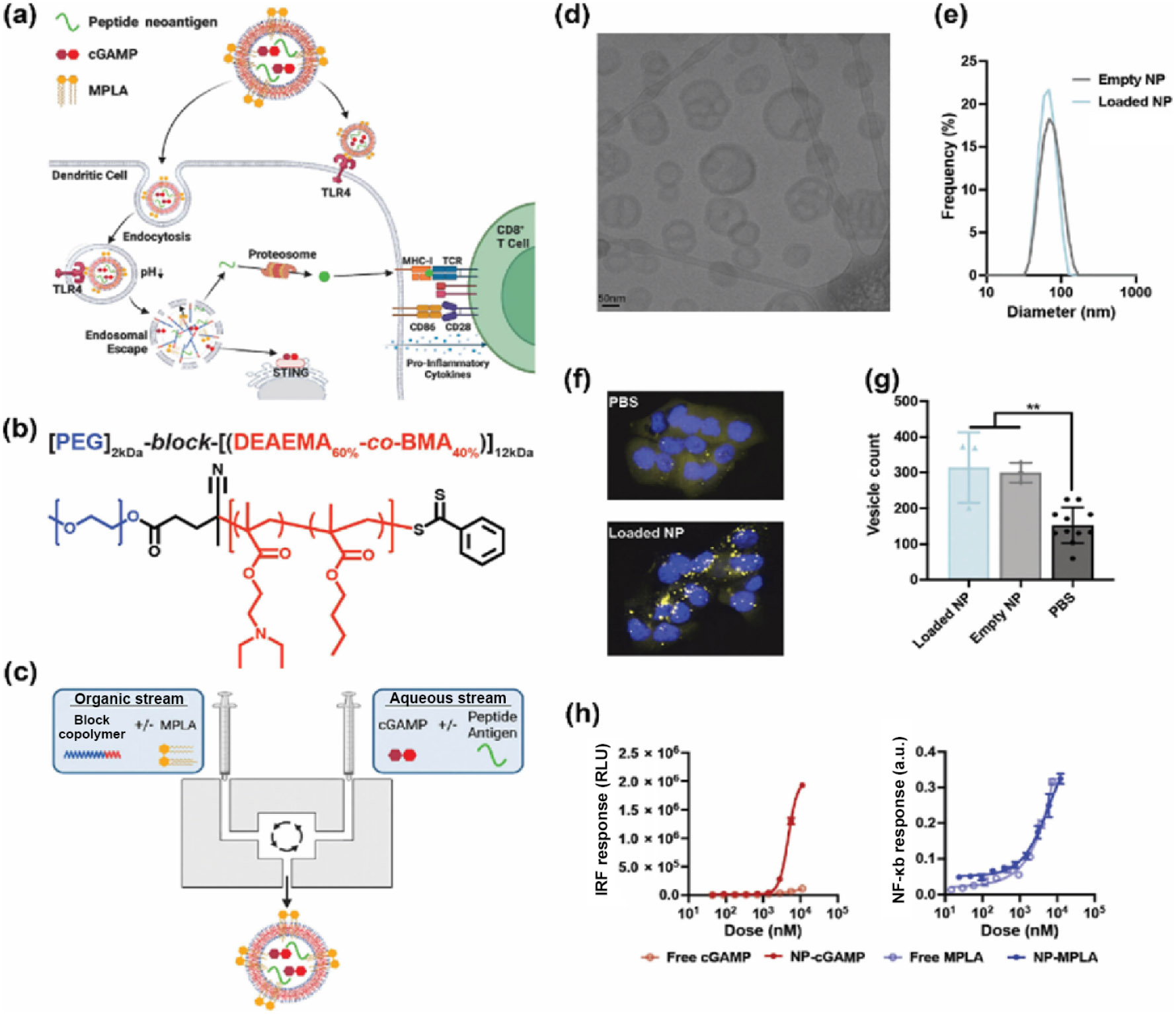
Development and characterization of nanovaccine. (a) Illustration of the nanoplatform. (b) Chemical structure of [PEG]2kDa-block-[(DEAEMA60%-co-BMA40%)] 12kDa. (c) Manufacturing of nanoparticles by confined impingement jet mixing. (d) Representative cryogenic electron micrograph of the nanoparticle. (e) Size characterization of empty nanoparticles and nanoparticles loaded with antigen, cGAMP, and MPLA. (f) Fluorescent images of NCI H358 with the treatment of PBS and loaded nanoparticles. (g) Vesicle count result of NCI H358 cells by Gal9-mCherry reporter assay. (h) Interferon regulatory factor 3 (IRF3) and NF-*κ*B activation evaluation after 24 h treatment [82]. Reproduced with permission from Ref. [82], © Baljon, J. J. et al. Published by American Chemical Society 2024.

In addition to FDA-approved polymers, novel structures have also been developed. Gong et al. designed a proton-driven nanotransformer-based vaccine (NTV) consisting of polymer-peptide conjugate p(DMAEMA 22 -OGEMA 4)- b-p(MAVE) 30 and antigenic peptide. In an acidic endosomal environment, the nanotransformer changes from nanospheres to nanosheets. Notably, NTV2 promoted DC maturation even in the absence of adjuvants. NTV2-induced bone marrow-derived myeloid dendritic cells (BMDCs) maturation occurred through activation of the NLRP3 inflammasome pathway, and NTV2 enhanced antigen processing in DCs. The antitumor effects of the nanotransformer were demonstrated across multiple tumor models, including *in vivo* tumor B16F10-OVA model, HPV-E6/E7 tumor model, and B16F10 neoantigen model [117]. Additionally, Chen's group developed a uniform polymeric nanoplatform based on poly(2-oxazoline)s (POx). The amphiphilic poly(2-oxazoline) (PMMEBOx) was conjugated with aTLR 7/8 agonist, and then neoantigen peptide was linked to PMMEBOx by click reaction. This POx-based nanovaccine platform efficiently loaded neoantigen peptides with varying physiochemical properties. The *ex vivo* fluorescence image demonstrated the promoted availability and delivery efficiency at lymph nodes. As a result, PMMEBOx-B-IMDQ exhibited remarkable antitumor efficacy in mouse models (Fig. 2) [113]. Furthermore, Baljon et al. recently developed a nanoparticle vaccine platform for co-delivery of peptide neoantigens and optimized combinations of STING and TLR4 agonists to enhance antigen-specific CD8^+^ T cell responses and improve the efficacy of immune checkpoint blockade in cancer immunotherapy (Fig. 3) [82].

In addition to liposome, polymeric nanoparticles have also been explored in clinical applications. Recently, Jeroen et al. developed a PLGA-based immunomodulating nanomedicine known as PRECIOUS-01. This formulation encapsulated the New York Esophageal Squamous Cell Carcinoma-1 (NY-ESO-1) cancer-testis antigen peptides along with alpha-galactosylceramide (*α*GalCer)-derived iNKT cell activator IMM60 (PRECIOUS-01). PRECIOUS-01 is currently undergoing a Phase 1 clinical trial, where its safety, tolerability, and efficacy will be evaluated in patients with advanced NY-ESO-1-expressing solid tumors [118, 119]. Additionally, a first-in-human dose escalation study of PRECIOUS-01 is underway [118].

As summarized in Table 6, polymeric nanoparticles hold significant clinical potential for enhancing cancer immunotherapy by improving antigen delivery and immune activation. Approved polymers like PLGA and innovative structures have shown promise in preclinical studies [113-117]. The ongoing clinical trials of formulations like PRECIOUS-01 further highlight the translational potential of polymeric nanoparticles in addressing unmet needs in cancer treatment, with anticipated results expected to provide valuable insights into their safety, efficacy, and broader applicability in oncology.

#### 3.2.3 Dendrimer nanoparticles

**Figure 4:**
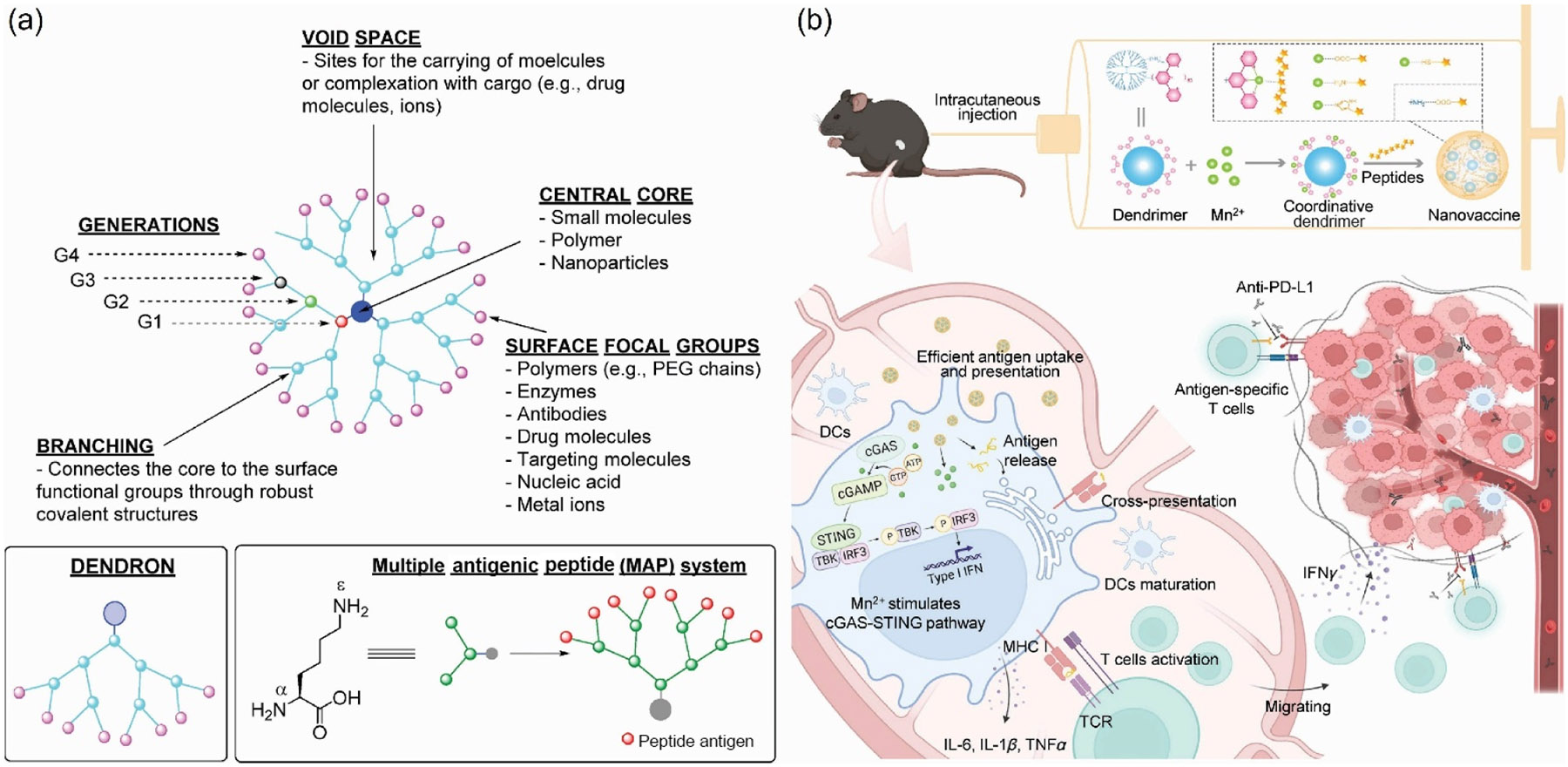
(a) Schematic illustration of dendritic structure. A dendrimer consists of a central core, void space, generations, branching, and surface functional groups [121]. Reproduced with permission from Ref. [121], © Published by Elsevier Ltd. All rights reserved 2021. (b) Schematic illustration of GT-Mn^2+^/OVA257-280. A fifthgeneration (G5) polyamidoamine (PAMAM) dendrimer was functionalized with 2,2':6',2"-Terpyridine-4'-carbaldehydes (TPys), followed by coordination with a variety of metal ions, including Mn^2+^, and then combined with an antigen peptide to produce the final vaccine formulation. This self-assembled nanovaccine process carrier and adjuvant function simultaneously [124]. Reproduced with permission from Ref. [124], © Elsevier Inc. 2023.

Dendrimer nanoparticles consist of the internal core and branched arms connecting the inner core with surface functional groups [120]. This highly branched scaffold gives dendrimers unique advantages exhibiting tremendous potential in cancer vaccines and making dendrimer an ideal adjuvant or carrier for cancer vaccines: (1) the external periphery functional groups offer flexibility for targeted modification, (2) the hyper-branched structure protects cargo from degradation, (3) the size and shape of dendrimer are precisely controllable, (4) the void space allows high encapsulate capacity (Fig. 4(a) [121].

Furthermore, the dendrimer also shows an adjuvanticity, allowing them to be combined with inactive antigenic peptides to create a self-adjuvating delivery system [121, 122]. For example, several polymer-peptide conjugates were designed as candidates against HPV-related cancers. Without the external help of adjuvant, polyacrylate star-polymer and epitope conjugates could demonstrate therapeutic effects after a single administrate, extensively overcoming the poor immunogenicity typically associated with peptide-based vaccines [123].

At present, no dendrimer nanoparticles are available in clinical trials as cancer vaccines. The potential reasons include the poor design of vaccine carriers and the complex manufacturing process [124]. In order to promote the clinical translation of dendrimer nanoparticles, Cao et al. reported a stable nanovaccine created by self-assembling dendrimers coordinated with Mn^2+^ with antigen peptide (GT-Mn^2+^/OVA257-280). GT-Mn^2+^/OVA257-280 was prepared using a simple method and can load multiple types of antigen peptides, enabling the creation of personalized vaccines. Their results demonstrated that the formulation extensively improved the cellular uptake of antigens and greatly promoted the maturation of DC and cross-presentation. Furthermore, GT-Mn^2+^/OVA257-280 elicited strong antigen-specific T-cell responses and effectively suppressed OVA-expressing melanoma growth (Fig. 4(b). The simple manufacturing process, versatility in encapsulating multiple neoantigens, excellent biocompatibility, and outstanding therapeutic efficacy make GT-Mn^2+^/OVA257-280 a promising candidate for clinical translation. Similarly, Zhang et al. engineered a facile multifunctional dendrimer peptide, KK2DP7, which not only targets lymph nodes but also functions as both an adjuvant and a delivery vehicle. This innovative KK2DP7 polymer nanovaccine system effectively delivered the OVA257–264 peptide to BMDCs, thereby enhancing antigen uptake. Leveraging dendritic cell transport, KK2DP7 navigated to the lymph nodes, stimulated dendritic cell maturation, and subsequently improved the cross-presentation of the KK2DP7/OVA complex. The researchers compared the efficacy of the novel adjuvant KK2DP7 with that of conventional adjuvants, including poly(I:C), CpG, and aluminum-based formulations. Animal studies revealed that the KK2DP7-based cancer vaccine demonstrated superior anticancer effects compared to those of traditional adjuvants. Moreover, when combined with anti-PD-1 antibodies, the KK2DP7-based cancer vaccine significantly prevented tumor recurrence in a postoperative model of recurrent tumors. The "multi-linkage" combination improved immune responses and the clinical efficacy of the antigen [125].

In summary, dendrimers are macromolecules with highly branched spherical structures, possessing well-defined chemical compositions, shapes, and sizes that can be precisely controlled (Table 7). Designing simple, universal, and multifunctional dendrimers is an effective strategy for clinical translation.

#### 3.2.4 Inorganic nanomaterials

Inorganic nanomaterials refer to nanomaterials that do not contain carbon. Unlike organic nanoparticles, inorganic nanomaterials have distinctive optoelectrical properties and superior stability [126, 127]. Notably, the large surface area to volume ratio of inorganic nanoparticles endows them with high loading capacity while maintaining chemical reactivity, so they are easier to functionalize with various ligands or targeting agents to produce more stable and lasting surface modifications, thereby achieving precise targeting and controlled release [127, 128]. Of all inorganic nanomaterials, silica-based and metallic-based materials are the most employed nanomaterials in cancer vaccines [129-134]. The prime examples of these two inorganic nanomaterials are mesoporous silicon and gold nanoparticles.

**Figure 5:**
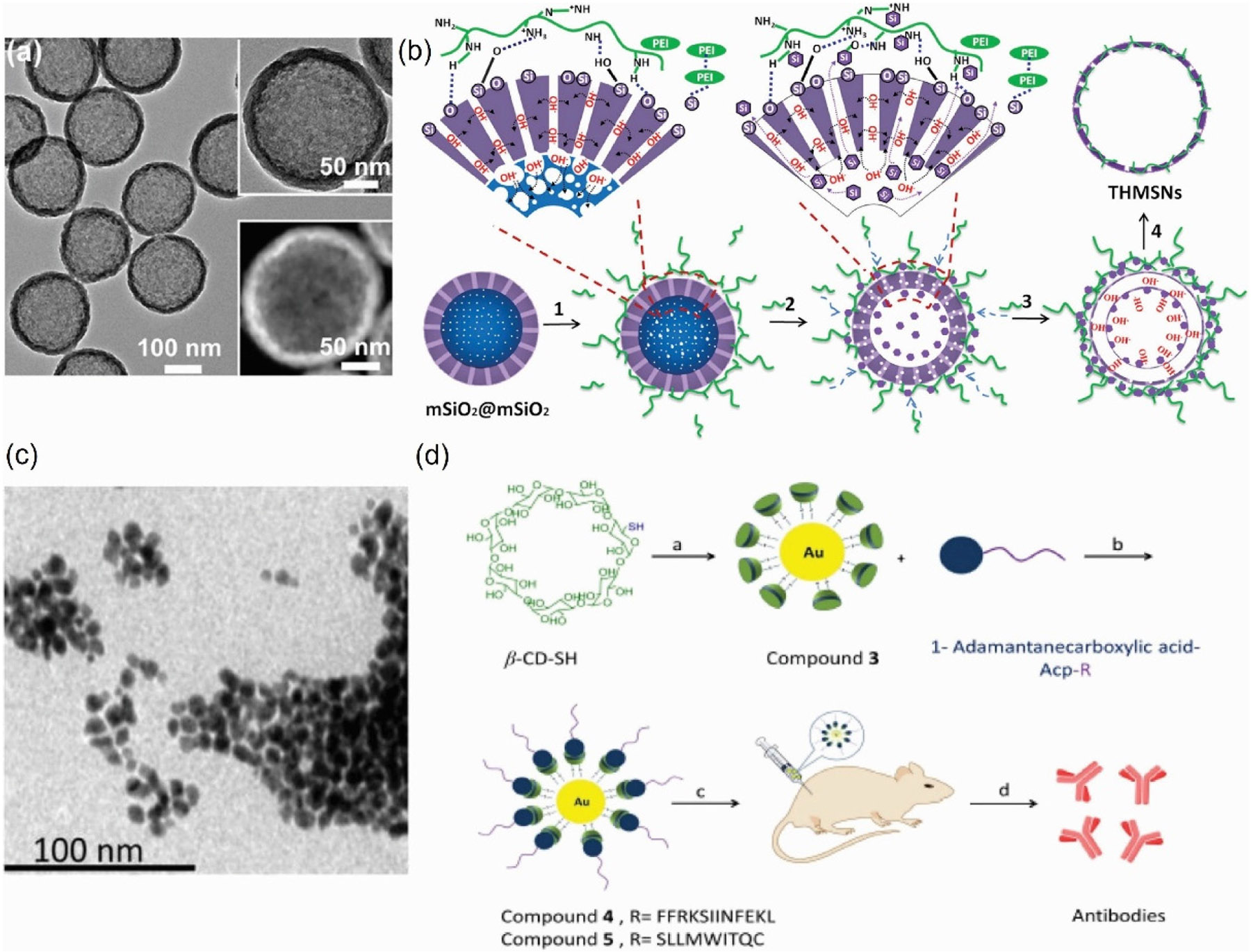
Fabrication illustration of THMSNs and non-covalent glycosylated gold nanovaccine. (a) TEM image of THMSNs. (b) The process involves adsorbing B-PEI25k onto mSiO2@mSiO2 NPs, etching and redepositing both the core and shell, and finally etching the NPs to form THMSNs [138]. Reproduced with permission from Ref. [138], © American Chemical Society 2019. (c) TEM image of compound 3. (d) The host system was synthesized by attaching *β*-CD to gold nanoparticles. The guest component was created by conjugating an antigen peptide with AD. The final formulation, Au-*β*-CD/AD-Acp-FFRKSIINFEKL, was achieved through selfassembly [145]. Reproduced with permission from Ref. [145], © Published by Elsevier B.V. on behalf of Chinese Chemical Society and Institute of Materia Medica, Chinese Academy of Medical Sciences 2019.

Mesoporous silicon is a type of silicon nanomaterial that has attracted widespread attention due to its unique pore structure [135]. To load an antigen or adjuvant into mesoporous silicon, a common method is through physical adsorption within the pores of the silicon nanoparticles [135]. Recently, Li et al. reported a straightforward adsorption method that incorporated the adjuvant polyethyleneimine (PEI) and antigen peptide into a mesoporous silica microrod (MSR–PEI), directly enhancing the immunogenicity of the peptide antigen. The vaccine could be assembled in under three hours by simply mixing all the ingredients and stored in lyophilized form before or after the addition of the antigen, simplifying the clinical application of the vaccine [136]. Hollow mesoporous silica nanoparticles (HMSN) have also demonstrated their ability to facilitate and enhance anti-tumor immunity and serve as an immune adjuvant in cancer therapy [137-139]. For example, Xie et al. fabricated monodisperse and stable hollow mesoporous silica nanoparticles enveloped with monophosphoryl-Lipid A (MPLA)-entrapped lipid bilayers (HTM@HMLB) for co-delivery of hydrophilic and hydrophobic peptides to enhance antitumor immune responses. It demonstrated that HTM@HMLB can significantly improve the stability of antigenic peptides and exhibit excellent biocompatibility. Furthermore, it promoted the activation and maturation of DCs and further induced the activation of antigen-specific CD4^+^ and CD8^+^ T-lymphocytes, extensively inhibiting tumor growth and melanoma lung metastasis [140]. In addition to their roles as delivery vehicles, HMSN exhibited self-adjuvanticity. When formulated into a novel cancer vaccine, they stimulated Th1 anti-tumor immunity *in vivo* and sustained immunological memory. Liu et al. prepared thin-shell PEI-hybrid HMSNs (THMSNs) with PEI etching strategy. THMSNs exhibited high drug loading capacity and sustained release profile. In an *in vitro* maturation of immature DCs study, they found that without the influence of PEI, the calcined THMSNs still showed enhanced expression of CD86 on DCs compared with calcined HMSNs, indicating that thin-shell structures promoted the maturation of DCs. Moreover, Trp2@THMSNs also showed a better effect in suppressing tumor growth in the tumor challenge model without any side effects (Figs. 5(a) and 5(b) [138].

For over a century, gold nanoparticles (AuNPs) have fascinated scientists with their versatile properties and are now emerging as promising candidates for therapeutical cancer vaccines [141-143]. Almeida et al. investigated the use of AuNPs as carriers for peptide cancer vaccines, specifically for delivering ovalbumin (OVA) peptide antigen and CpG adjuvant. Their AuNP-peptide vaccine induced strong antigen-specific responses and anti-tumor activity in preventive and therapeutic tumor models, indicating their potential as effective vaccine carriers that allowed for lower and safer doses of other adjuvants [144]. Additionally, Liao group developed a non-covalent glycosylated gold nanoparticles/peptides nanovaccine composed of two components: a host system synthesized by attaching *β*-cyclodextrin (*β*-CD) to gold nanoparticles and a guest system consisting of 1-Adamantanecarboxylic acid (AD) combined with the antigen peptide. This nanovaccine enhanced immune responses and anticancer effects in melanoma through non-covalent interactions between host and guest systems, making it a versatile platform for delivering various types of antigens, especially T cell-independent antigens, and offering a promising new approach for personalized vaccines (Figs. 5(c) and 5(d) [145].

Overall, the key findings from preclinical studies are summarized in Table 8. Although no inorganic nanoparticles for peptide-based cancer vaccines are currently available in clinical trials, the promising results from preclinical studies — such as enhanced stability, improved safety profiles, and simplified manufacturing processes — highlight the potential for clinical translation.

### 3.3 Toxicity concern of nanoadjuvants

Nanoadjuvants offer significant potential for enhancing the efficacy of vaccines and drug delivery systems, but their toxicity must be carefully managed to ensure safety in clinical applications. The toxicity of nanoadjuvants is influenced by several factors, including size, shape, material composition, surface charge, and functionalization. To achieve lymphatic uptake and access the lymph nodes, the desired size range is between 10 and 100 nm [146]. However, smaller nanoparticles can penetrate cells and tissues, potentially accumulating in organs such as the liver and lung, and may cause toxicity. The material composition of nanocarriers also affects their toxicity profile, with inorganic materials like silica or silver nanoparticles exhibiting higher cytotoxicity compared to biocompatible polymers. Takao et al. point out that oral administration of small SiO_2_ nanoparticles exacerbated intestinal inflammation [147]. In addition, some metal-based nanoparticles, like Ag and ZnO, were shown to bind to estrogen receptors and demonstrate estrogenic activity. This can disrupt endocrine balance, particularly in pregnant women and children [148]. Surface charge and functionalization play crucial roles in determining cellular interactions and immune responses, with positively charged nanoparticles more likely to cause toxicity, including hepatotoxicity and pro-inflammatory responses, due to stronger interactions with negatively charged cell membranes, which potentially disrupt cellular functions and trigger inflammatory pathways [149].

To mitigate the potential toxicity of nanoadjuvants, several strategies can be employed. Surface modification, such as PEGylation, can reduce immune recognition and prevent aggregation, thereby minimizing toxicity [150]. Optimizing the size and shape of nanoparticles to enhance clearance and reduce tissue accumulation is also essential [151]. Using biodegradable materials ensures that nanoparticles break down into non-toxic byproducts, which are safely eliminated from the body [152, 153]. Combining nanoadjuvants with other therapeutic modalities can lower the required dose of each agent, further decreasing toxicity [154]. Thorough preclinical testing and long-term monitoring are crucial for evaluating the safety and efficacy of nanoadjuvants, ensuring that they are biocompatible and effectively cleared from the body.

## 4 Clinical status of nanotechnology-based adjuvants

The details of the clinical study of the therapeutic cancer vaccine are summarized in Table 9. According to Table 9, most of the nanovaccines in clinical trials are liposomes. Up to now, a total of two vaccines, L-BLP25 and PDS0101, have entered Phase 3 clinical trials, and the others are in early clinical trials.

DPX-0907 is a novel liposomal cancer vaccine composed of seven HLA-A2-restricted peptides derived from tumor-associated antigens, along with a universal T-helper peptide, all formulated using the DepoVax^™^ liposome-in-oil platform. DepoVax^™^ efficiently encapsulated antigen peptides and adjuvants in the oil medium without the need for emulsification, simplifying the clinical practice of oil-based vaccines. In a Phase 1 first-in-man clinical trial, DPX-0907 demonstrated a favorable safety profile, with no dose-limiting toxicities and mild injection site reactions. The vaccine induced antigen-specific T cell responses in 61% of patients, with rapid and sustained immune activation, particularly in breast and ovarian cancer patients. Immune responses were more frequent in these patients compared to those with prostate cancer, likely due to differences in disease progression or prior treatments. Despite these variations, the findings suggest that DPX-0907 may be more effective in patients with stable disease or those who have undergone fewer prior treatments. Overall, DPX-0907 shows promising safety and immunogenicity, making it a strong candidate for further clinical trials. Future studies will optimize patient selection and explore the incorporation of immune modulators to enhance the vaccine’s efficacy against micrometastatic disease [155].

ONT-10 is liposome formulated with a synthetic glycopeptide antigen from human MUC1 [156]. In the completed Phase 1 clinical study, ONT-10 was well tolerated, with all treatment-related adverse events classified as Grade 1 or 2. MUC1-specific immune responses were observed in the majority of patients, and 68% demonstrated encouraging disease control [157, 158].

The investigational therapeutic cancer vaccine L-BLP25 is a lyophilized liposome consisting of tecemotide, immunoadjuvant monophosphoryl lipid A, and lipids cholesterol, dimyristoyl phosphatidylglycerol (DMPG), and dipalmitoyl phosphatidylcholine (DPPC). The Phase 1 studies in NSCLC patients demonstrated minimal toxicity with vaccination. Two Phase II trials established the optimal dose and regimen of L-BLP25, demonstrating that the vaccine elicited a T-cell proliferative response. In a randomized Phase II study in NSCLC, the median overall survival was 30.6 months for patients treated with L-BLP25, compared to 13.3 months for those receiving best supportive care. Although this was a subgroup analysis with a non-significant *P* value, the observed survival benefit of L-BLP25 supported the need for further investigation [159]. However, in Phase III studies, L-BLP25 did not significantly improve overall survival compared to placebo [100, 160-162].

The progress of PDS0101 has been discussed in the liposome part. PDS0101 is the most promising candidate for clinical translation. Right now, it is under Phase 3 clinical trial with an estimated completion date in December 2025 [105, 163].

Though the nanotechnology-based adjuvants exhibit a promising trend in the preclinical and clinical studies, to date, no peptide-based therapeutic cancer nanovaccine has been approved by the US FDA. With the conversion rate from basic scientific research to clinical application being less than 10%, the clinical translation and commercialization of nanomedicines face numerous hurdles [164]. First, the biggest challenge in clinical studies is the difficulty in recruiting and retaining eligible participants. For example, the clinical trial NCT05232851, which began in 2022, is still in the recruitment phase. Some vaccines that demonstrated outstanding outcomes in Phase I studies have failed to advance to Phase II or III trials for decades. To address this issue, automating clinical trial software would help screen eligible patients [165, 166]. Recently, the trusted Cloud-based application has been developed to assist in data collection. The promising results showed that the trusted Cloud-based application improved the data accuracy and quality moved from electronic health record (EHR) systems while saving time for clinical research [167]. Additionally, a lack of transparency poses a significant challenge. Due to strict privacy regulations surrounding medical records, the results of completed clinical trials often remain undisclosed and unpublished for decades [168]. To address this issue, policies must be streamlined, clarified, and revised to ensure greater accountability and data accessibility.

Overall, nanoadjuvants such as PDS0101 showed promising potential in clinical trials. However, challenges including difficulties in recruiting and retaining eligible participants, and a lack of transparency continue to hinder the clinical translation of nanovaccines. The integration of automation technologies, along with the establishment and refinement of supportive policies, will be critical in accelerating the translation process.

## 5 Disscussion

This review summarizes the adjuvants for therapeutic peptide-based cancer vaccines, with a particular focus on nanoadjuvants. Numerous studies have demonstrated the significant potential of intelligent nanoplatforms to enhance the safety and efficacy of peptide-based cancer vaccines in preclinical and clinical models. From a clinical perspective, nanoadjuvants offer pioneer solutions to the challenge in the fight against cancers. The benefits of targeted drug delivery, safeguarding of peptide antigens, sustained release profile, adjuvant function, and minimal systemic toxicity prove them the “Seed players” for clinical translations. However, challenges remain in translating promising preclinical results into effective and safe clinical applications. One potential reason is the excessive emphasis on multifunctional and versatile nanosystems. The complex synthesis and preparation processes pose significant challenges for large-scale production, thereby hindering the clinical translation of nanomedicines. Therefore, to facilitate the clinical transformation of nanomedicines, the design of nanovaccines should be simplified while still addressing clinical needs [180]. Another approach to addressing these challenges is the adoption of Quality by Design (QbD), a strategy recommended by the FDA and the International Conference on Harmonization (ICH) for nanomedicine design, manufacturing, and regulation [181]. When applying QbD at the early stages of research, critical quality attributes — such as the route of administration, dosage, biological characteristics of the vaccine recipient, and process parameters — along with the quality of the final product, are carefully considered. This approach provides essential principles for the successful commercialization of nanomedicines [182, 183].

Another significant challenge for clinical translation is the technology itself. Traditional nanomedicine preparation methods are time-consuming and labor-intensive, making large-scale manufacturing impractical. However, emerging technologies like microfluidic technology and particle replication in non-wetting template (PRINT) technology offer solutions for the controllable, reproducible nanomedicine production on a large scale [184, 185]. Furthermore, the significant differences between preclinical animal models and human patients remain a key obstacle to clinical success. While subcutaneous mouse models are widely used to evaluate the safety and efficacy of nanotherapeutics, they do not accurately replicate human tumor conditions, which contributes to the failure of clinical trials [186]. Recently, more and more orthotopic models, patient-derived xenografts, and genetically engineered mouse models (GEMMs) have been developed, making it possible to stimulate the complexity and heterogeneity of human tumors to a high degree [187, 188]. Additionally, with the rapid development of artificial intelligence (AI), data-driven models can also be powerful in the prediction of human tumors with accuracy [189, 190].

Furthermore, the regulatory challenges should also be addressed. Until now, most of the nanomedicine approvals have been based on conventional regulations, which are less suited for the nanovaccine clinical translation due to the significant differences in manufacturing processes compared with traditional formulations, such as tablets and capsules. As shown in Table 9, most of the nanovaccines are liposomes, likely due to the existence of specific guidelines for liposomal products. However, these guidelines are limited to PK/PD-modifying liposomes and do not apply to other systems [164]. Therefore, it is crucial to establish globally standardized regulations.

In summary, this review highlights the potential of nanoadjuvants to enhance the safety and efficacy of peptide-based cancer vaccines. However, clinical translation is hindered by complex nanosystems and production challenges. Simplifying design and adopting QbD principles can address these issues. Emerging technologies like microfluidics and PRINT offer scalable production solutions. Advanced preclinical models and AI can improve human tumor prediction accuracy. Lastly, developing globally standardized regulations is critical for the successful clinical translation of nanovaccines.

## Figures and Tables

**Table 1:** Summary of clinical trials with TLR agonists for peptide-based cancer vaccine

ID No.	Cancer	TLR	Adjuvants	Stage	Recruitment status	Ref.
NCT04364230	Melanoma	TLR3	Poly ICLC	Phase 1/2	Completed	[35]
NCT01532960	Breast cancer	TLR3	Poly ICLC	Phase 1	Terminated	[36]
NCT01585350	Melanoma	TLR3	Poly ICLC, Lipopolysaccharide, Montanide ISA-51	Phase 1	Completed	[27, 37]
NCT02320305	Melanoma	TLR4	Glucopyranosyl lipid A	Early phase 1	Completed	[38]
NCT00960752	Melanoma	TLR7	Resiquimod (R848)	Phase 2	Completed	[39]
NCT00669292	Esophageal cancer	TLR9	Montanide ISA 51, CpG7909	Phase 1/2	Unknown status	[40]
NCT01264731	Melanoma	TLR7	Imiquimod	Phase 1	Completed	[41]
NCT00470379	Melanoma	TLR7	Resiquimod	Early phase 1	Completed	[42]
NCT01748747	Melanoma	TLR7	Resiquimod	Early phase 1	Completed	[43]
NCT02126579	Melanoma	TLR3, TLR7	Poly ICLC, Resiquimod, Montanide ISA-51	Phase 1/2	Completed	[44]

**Table 2:** Summary of the mechanism and clinical status of immunopotentiators

Adjuvant	Mechanism of action	Clinical status	Ref.
IFA	Emulsifies antigens in mineral oil to enhance immune response.	Widely used in animal studies; not approved for human use due to toxicity.	[25]
TLRs agonists	Recognize PAMPs or DAMPs to activate immune responses.	Approved for human use in antiviral and skin cancer treatments (Imiquimod).	[17]
NLRs agonists	Intracellular receptors that detect cytosolic PAMPs and DAMPs result in the activation of inflammatory cytokines and/or chemokines.	Under investigation for cancer immunotherapy.	[47]
STING agonists	Activates the cGAS-STING pathway, leading to type I interferon production and enhanced antiviral and antitumor responses.	Under investigation for cancer immunotherapy.	[48, 51]
IL-2	Enhances T cell proliferation, differentiation, and survival.	Approved for melanoma and renal cell carcinoma.	[60–62]
GM-CSF	Activates monocytes/macrophages and enhances DC differentiation.	Under investigation for cancer immunotherapy.	[63]

**Table 3:** The advantages of nanotechnology-based adjuvants

Advantage	Nanotechnology-based adjuvants	Conventional adjuvants	Ref.
Targeted delivery	Precise delivery to tumor sites or lymph nodes, reducing off-target effects, and lowering the risk of toxicity and side effects	General distribution with involuntary systemic exposure, yielding non-specific adverse effects	[72–74]
Controlled release	Sustained, controlled release of antigens and adjuvants for a prolonged immune response	Rapid release, often leading to short-lived immune activation	[75–77]
Enhanced stability	Protects antigens and conventional adjuvants from degradation, maintaining their potency	Susceptible to degradation prior to stimulating the immune system	[78]
Modulation of TME	Helps convert cold tumors to hot tumors by overcoming the immunosuppressive TME	Does not specifically address or modify the TME	[79]
Customization of administration routes	Flexible routes (e.g., inhaled, topical) tailored to patient needs	Typically administered by injection or other invasive routes	[85–87]
Synergistic therapies	Synergizes with other therapies (e.g., chemotherapy, immunotherapy) for enhanced efficacy via synchronizing the co-delivery of different payloads and enables the tumor colocalization	Limited synergy with other therapies due to failing to achieve the co-delivery to tumors; primarily used as standalone agents	[81–83]
Higher patient compliance	Reduced dosing frequency and non-invasive options improve patient adherence	Requires more frequent injections and invasive methods	[88]
Self-adjuvants	Some nanomaterials act as self-adjuvants, directly enhancing the immune response	Typically requires external adjuvants to boost the immune response	[88]

**Table 4:** Summary of preclinical studies on liposomal nanovaccines

Category	Components	Clinical limitations	Key findings	Ref.
Liposomebased vaccines	• Antigen: HPV16 E7 SLP • Lipid: DOTAP and DOPC	• Adverse events associated with Montanide • Weak efficacy in end-stage HPV16^+^ cervical cancer	• No adverse effects at the injection site • High frequency of antigen-specific CD8^+^T cells • Outstanding efficacy in eradicating large HPV16^+^ tumors	[94]
	• Antigen: P5 peptide • Immunostimulator: MPL • Lipid: DMPC, DMPG, DOPE, Cholesterol, Maleimide-PEG2000-DSPE	• Systemic toxicity	• Safe use in humans • Easy manufacturing process • Higher IFN-*γ* production by CD8^+^ T cells compared to other formulations, indicating a robust CTL response • Superior inhibition of tumor growth and prolonged survival in TUBO tumor mice model	[95]
	• Antigen: A5 peptide • Immunostimulator: QS-21, PHAD • Lipid: CoPoP	• Insufficient generation of antigenspecific CD8^+^ T cells	• Formation of well-tolerated and stable vaccine • Robust induction of antigen-specific CD8^+^ T cell respons • Capability to control both local and metastatic disease	[96]

**Table 5:** Clinical trials of PDS010

ID No.	Intervention	Stage	Status	Disease	Ref.
NCT02065973	PDS0101	Phase 1	Completed	Cervical intraepithelial neoplasia (CIN) and HPV infection	[102]
NCT04287868	PDS0101 M7824 NHS-IL12	Phase 1 Phase 2	Active, not recruiting	Advanced HPV associated malignancies	[109]
NCT05232851	PDS0101, Pembrolizumab	Phase 1 Phase 2	Recruiting	HPV-oropharyngeal squamous cell carcinoma (HPVOPSCC)	[110]
NCT04708470	PDS0101 Bintrafusp Alfa Entinostat	Phase 1 Phase 2	Active, not recruiting	HPV-associated malignancies, small bowel, and colon cancers	[111]
NCT04260126	PDS0101 Pembrolizumab (KEYTRUDA^®^)	Phase 2	Active, not recruiting	Recurrent/metastatic HPV16-positive head and neck squamous cell carcinoma (HNSCC)	[112]
NCT04580771	PDS0101 cisplatin radiation therapy	Phase 2	Active, not recruiting	Stage IB3-IVA cervical cancer	[107]
NCT06790966	PDS0101, Pembrolizumab	Phase 3	Recruiting	HPV16^+^ recurrent/metastatic head and neck squamous cell carcinoma	[108]

**Table 6:** Applications of polymeric nanoparticles in preclinical and clinical studies

Category	Examples	Key findings	Stage	Ref.
Polymeric nanoparticles	ATP + E7-NPs	• PLGA nanoparticles protected peptides from degradation, thereby significantly promoting sustained antigen uptake by DCs and migration to lymph nodes; • APT, as a new adjuvant, significantly enhanced the immunogenicity of PLGA nanoparticles by promoting DCs migration, maturation, and the priming and full polarization of CD8^+^ T cells.	Preclinical	[116]
	NTV	• No substantial systemic cytotoxicity; • A proton-driven nanotransformer-based vaccine transited from nanospheres to nanosheets in an acidic environment, disrupting the endosomal membrane and releasing antigens into the cytosol, thereby facilitating cross-presentation to CD8^+^ T cells; • NTV2 activated the NLRP3-inflammasome pathway to further improve antigen processing in DCs; • NTV2 inhibited tumor growth and extended survival in tumor-bearing mice.	Preclinical	[117]
	PMMEBOx vaccine	• Neoantigen peptides with various physiochemical properties could be loaded into PMMEBOx with the same efficacy by the same conjugation reaction, significantly lowering the manufacturing difficulties; • PMMEBOx nanovaccines increased the accumulation and retention of antigens in lymph nodes, thereby improving immunostimulation and antigen presentation for peptide antigens; • PMMEBOx nanovaccines improved the solubility of antigens and exhibited excellent generalizability in superior antitumor efficacy.	Preclinical	[113]
	NP-cGAMP/MPLA	• NP-cGAMP/MPLA enhanced lymph node accumulation and uptake by antigen-presenting cells.	Preclinical	[82]
	PRECIOUS-01	• No results posted.	Clinical	[118, 119]

**Table 7:** Summary and clinical translation potential of dendrimer nanoparticles

Category	Description	Key findings	Ref.
Dendrimer nanoparticles	Self-assembling dendrimers coordinated with Mn^2+^ and antigen peptide (GT-Mn^2+^/OVA257-280)	• GT-Mn^2+^/OVA257-280 improved cellular uptake, DC maturation, and antitumor effects in OVA-expressing melanoma; • GT-Mn^2+^ could encapsulate multiple neoantigens, enabling the creation of effective "personalized" cancer vaccines with a simple production process; • When combined with anti-PDL1, the GT-Mn^2+^/OVA257-280 formulation demonstrated excellent biocompatibility and therapeutic efficacy in melanoma, showing significant potential for clinical translation.	[124]
	Multifunctional dendrimer peptide (KK2DP7) as adjuvant and delivery vehicle	• The multifunctional dendrimer peptide (KK2DP7), with a simple preparation method, combined adjuvant and delivery vehicle functions targeting the lymph nodes, demonstrated superior anticancer effects and immune responses.	[125]

**Table 8:** Summary of preclinical studies of inorganic nanoparticle

	Example	Key findings	Ref.
Inorganic nanomaterials	MSR–PEI	• The vaccine could be assembled in less than 3 h by simply mixing all ingredients and stored in lyophilized form before or after the addition of antigen; • MSR–PEI eradicated local tumors and lung metastases and established immune memory in three models.	[136]
	HTM@HMLB	• Improved nanoparticle stability and biocompatibility; • Promoted DC activation and antitumor immune responses.	[140]
	THMSNs	• Enhanced antigen-loading efficacy and sustained drug release profiles; • No significant side effects; • Remarkably enhanced anticancer immune responses.	[138]
	AuNP-OVA	• AuNP-OVA was sufficient to provoke antigen-specific responses and anti-tumor activity.	[144]
	Non-covalent glycosylated gold nanoparticles/peptides nanovaccine	• Novel non-covalent interactions between nanoparticles and antigens.	[145]

**Table 9:** Therapeutic peptide-based cancer nanovaccine in clinical trials

Carrier	Name	ID No.	Stage	Status	Cancer	Ref.
Liposome	DPX-0907	NCT01095848	Phase 1	Completed	HLA-A2 positive advanced stage ovarian, breast, and prostate cancer	[97]
Liposome	ONT-10	NCT02270372	Phase 1	Completed	Advanced ovarian or breast cancer	[39]
Liposome	Lipovaxin-MM	NCT01052142	Phase 1	Completed	Melanoma	[99]
Liposome	L-BLP25	NCT00960115	Phase 1 Phase 2	Completed	NSCLC	[170]
		NCT01731587	Phase 1	Withdraw	NSCLC	[171]
		NCT01507103	Phase 2	Completed	Rectal cancer	[171]
		NCT01496131	Phase 2	Completed	Prostate Cancer	[101]
		NCT00157196	Phase 2	Completed	NSCLC	[171]
		NCT01094548	Phase 2	Completed	Multiple myeloma	[172]
		NCT01462513	Phase 2	Completed	Colon carcinoma	[173]
		NCT00157209	Phase 2	Completed	NSCLC	[174]
		NCT00828009	Phase 2	Completed	NSCLC	[175]
		NCT00925548	Phase 3	Terminated	Advanced breast cancer	[176]
		NCT01015443	Phase 3	Terminated	NSCLC	[161]
		NCT00409188	Phase 3	Completed	NSCLC	[100, 162]
		NCT02049151	Phase 3	Terminated	NSCLC	[177]
Liposome	PDS0101	NCT02065973	Phase 1	Completed	Cervical intraepithelial neoplasia (CIN) and HPV infection	[102]
		NCT04287868	Phase 1 Phase 2	Active, not recruiting	Advanced HPV associated malignancies	[109]
		NCT05232851	Phase 1 Phase 2	Recruiting	HPV-oropharyngeal squamous cell carcinoma (HPV-OPSCC)	[110]
		NCT04708470	Phase 1 Phase 2	Active, not recruiting	HPV-associated malignancies, small bowel, and colon cancers	[111]
		NCT04260126	Phase 2	Active, not recruiting	Recurrent/metastatic HPV16-positive head and neck squamous cell carcinoma (HNSCC)	[112]
		NCT04580771	Phase 2	Active, not recruiting	Stage IB3-IVA cervical cancer	[107]
		NCT06790966	Phase 3	Recruiting	HPV16^+^ recurrent/metastatic head and neck squamous cell carcinoma	[108]
Amphiphile	ELI-002 7P	NCT05726864	Phase 1 Phase 2	Active, not recruiting	Metastatic breast cancer	[178]
Polymeric nanoparticle	CHP-NY-ESO-1 (IMF-001)	NCT01003808	Phase 1	Completed	Esophageal cancer	[179]
